# Supplementary material for: Detailed Characterization of Small Extracellular Vesicles from Different Cell Types Based on Tetraspanin Composition by ExoView R100 Platform
Source: Int J Mol Sci. 2022 Aug 1;23(15):8544. doi: 10.3390/ijms23158544 (PMC9369185; doi:10.3390/ijms23158544)
Supplement: Supplementary file 1 [file ijms-23-08544-s001.zip › 220727_Supplementary information_proof_MS.pdf]

Supplementary information

Detailed Characterization of Small Extracellular Vesicles from Different Cell Types Based on Tetraspanin Composition by ExoView R100 Platform

Kai Breitwieser [1,†], Leon F. Koch [1,†], Tobias Tertel [2], Eva Proestler [1], Luisa D. Burgers [3], Christoph Lipps [4], James Adjaye [5], Robert Fürst [3], Bernd Giebel [2] and Meike J. Saul [1,*]

[1] Fachbereich Biologie, Technische Universität Darmstadt, 64287 Darmstadt, Germany; breitwieser@bio.tu-darmstadt.de (K.B.); leon.koch@tu-darmstadt.de (L.F.K.); ewolf@bio.tu-darmstadt.de (E.P.)
[2] Institute for Transfusion Medicine, University Hospital Essen, University of Duisburg-Essen, 45122 Essen, Germany; tobias.tertel@uk-essen.de (T.T.); bernd.giebel@uk-essen.de (B.G.)
[3] Institute of Pharmaceutical Biology, Goethe University Frankfurt, 60438 Frankfurt am Main, Germany; burgers@em.uni-frankfurt.de (L.D.B.); fuerst@em.uni-frankfurt.de (R.F.)
[4] Department of Internal Medicine I, Cardiology, Justus-Liebig-University Gießen, 35392 Gießen, Germany; christoph.lipps@gmail.com
[5] Institute for Stem Cell Research and Regenerative Medicine, Universitätsklinikum Düsseldorf, 40225 Düsseldorf, Germany; james.adjaye@med.uni-duesseldorf.de
* Correspondence: saul@bio.tu-darmstadt.de; Tel.: +49-(0)-6151-16-22005
† These authors contributed equally to this work.

A

Experimental event distribution

avg. particle count / capture spot

	CD63	CD81	CD9
non coloc	22.73	2.01	24.02
CD63/CD81	3.25	5.27	n.a.
CD63/CD9	17.71	n.a.	13.50
CD81/CD9	n.a.	37.96	26.26
CD63/CD81/CD9	56.31	54.76	36.22

Individual event probability

independent probability / capture spot

	CD63	CD81	CD9
CD63	1.00	0.60	0.50
CD81	0.60	1.00	0.62
CD9	0.74	0.93	1.00
no CD63	0.00	0.40	0.50
no CD81	0.40	0.00	0.38
no CD9	0.26	0.07	0.00

B

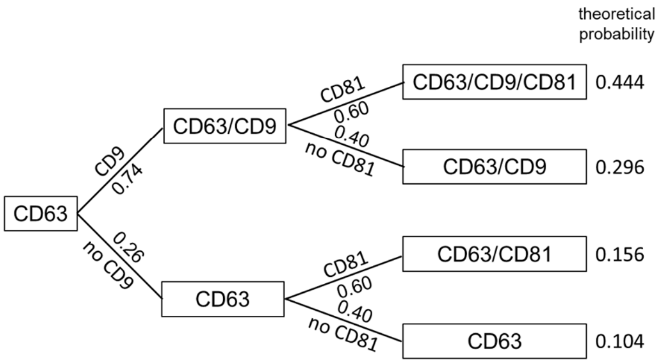

Figure S1. Exemplary calculation of the assessment of tetraspanin randomness. (**A**) Exemplary data transformation that showcases the extraction of the probability for an individual event. (**B**) Exemplary reconstruction of unbiased, expected tetraspanin colocalization probabilities.

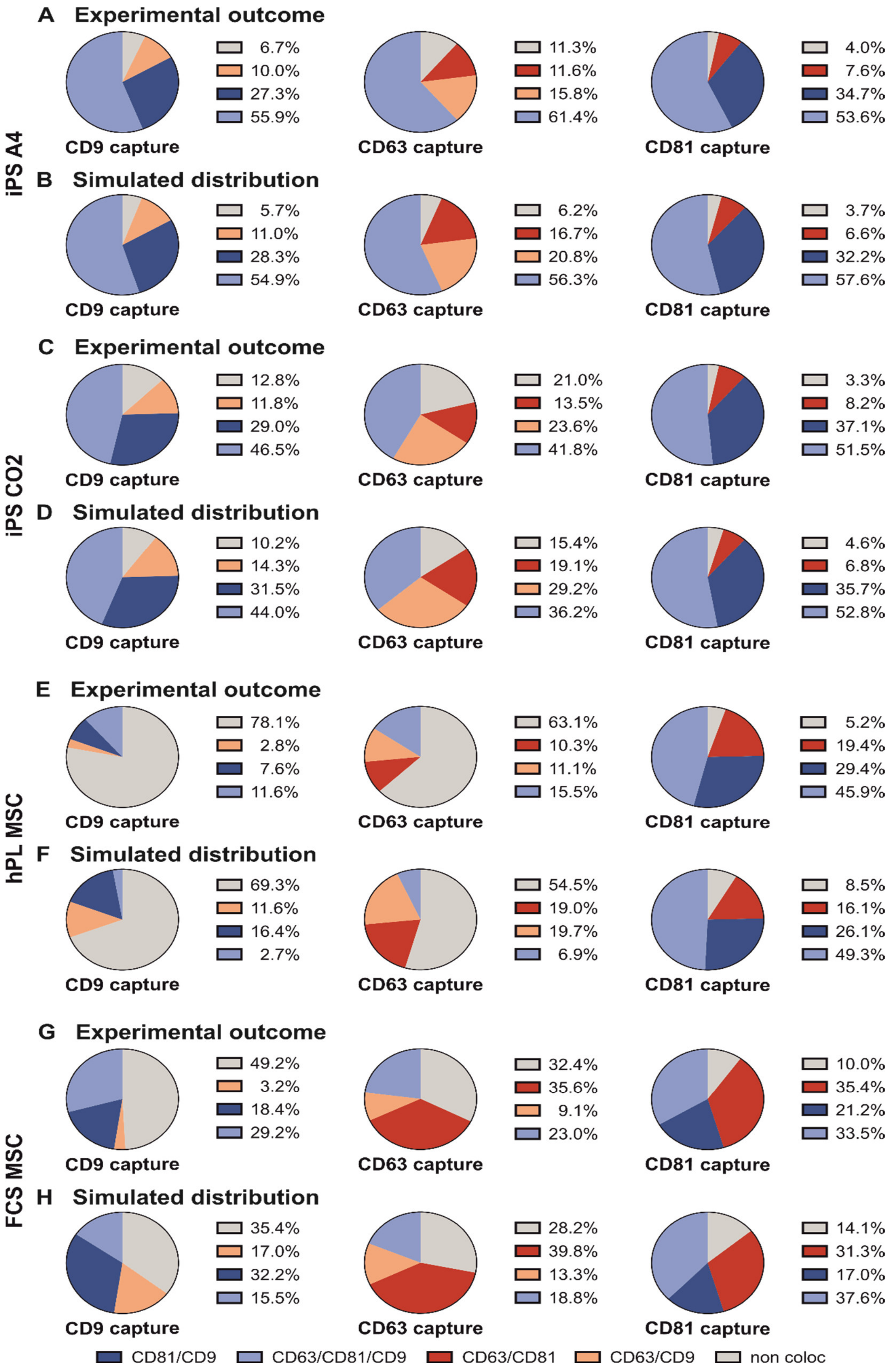

Figure S2. Differences in tetraspanin colocalization between experimental outcome and calculated random distribution of sEV derived from stem cells. (**A+C+E+G**) Experimental outcome of tetraspanin colocalization on the CD9/CD63/CD81 tetraspanin capture spots of hiPS A4- (**A**), hiPS C02- (**C**), hPL cultivated MSC- derived (**E**) and FCS cultivated MSC-derived (**G**) sEV using the ExoView R100 platform and fluorescent antibodies. (**B+D+F+H**) Simulated random tetraspanin colocalization using decision tree calculation based on experimentally determined data of hiPS A4-

(**B**), hiPS C02- (**D**), hPL cultivated MSC- derived (**F**) and FCS cultivated MSC-derived (**H**) sEV. Experimental data shown represents the respective tetraspanin colocalization fraction [%] out of all detected sEV. Plotted is the mean of three independent biological with three technical replicates, respectively. Predicted random distribution was calculated from the respective experimental data and was generated as indicated.

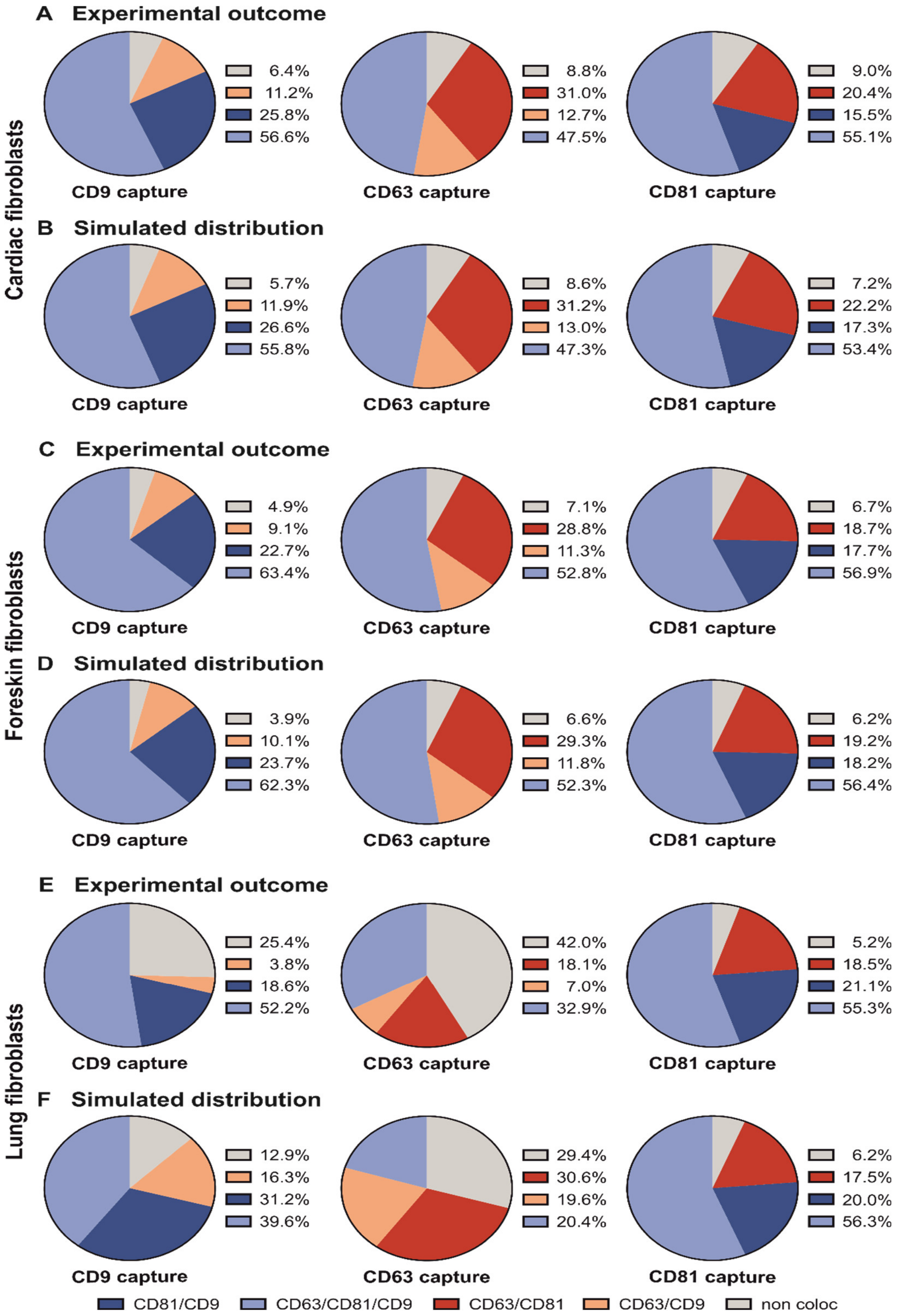

Figure S3. Differences in tetraspanin colocalization between experimental outcome and calculated random distribution of sEV derived from fibroblasts. (**A+C+E**) Experimentally determined tetraspanin colocalization on the CD9/CD63/CD81 tetraspanin capture spots of cardiac fibroblast- (**A**),

foreskin fibroblast- (**C**) and lung fibroblast- derived (**E**) sEV using the ExoView R100 platform and fluorescent antibodies. (**B+D+F**) Simulated random tetraspanin colocalization using decision tree calculation based on experimentally determined data of cardiac fibroblast- (**B**), foreskin fibroblast- (**D**) and lung fibroblast-derived (**F**) sEV. Experimental data are represented as mean of three independent biological experiments with three technical replicates. Predicted random distribution was calculated from the respective experimental data and was generated as indicated.

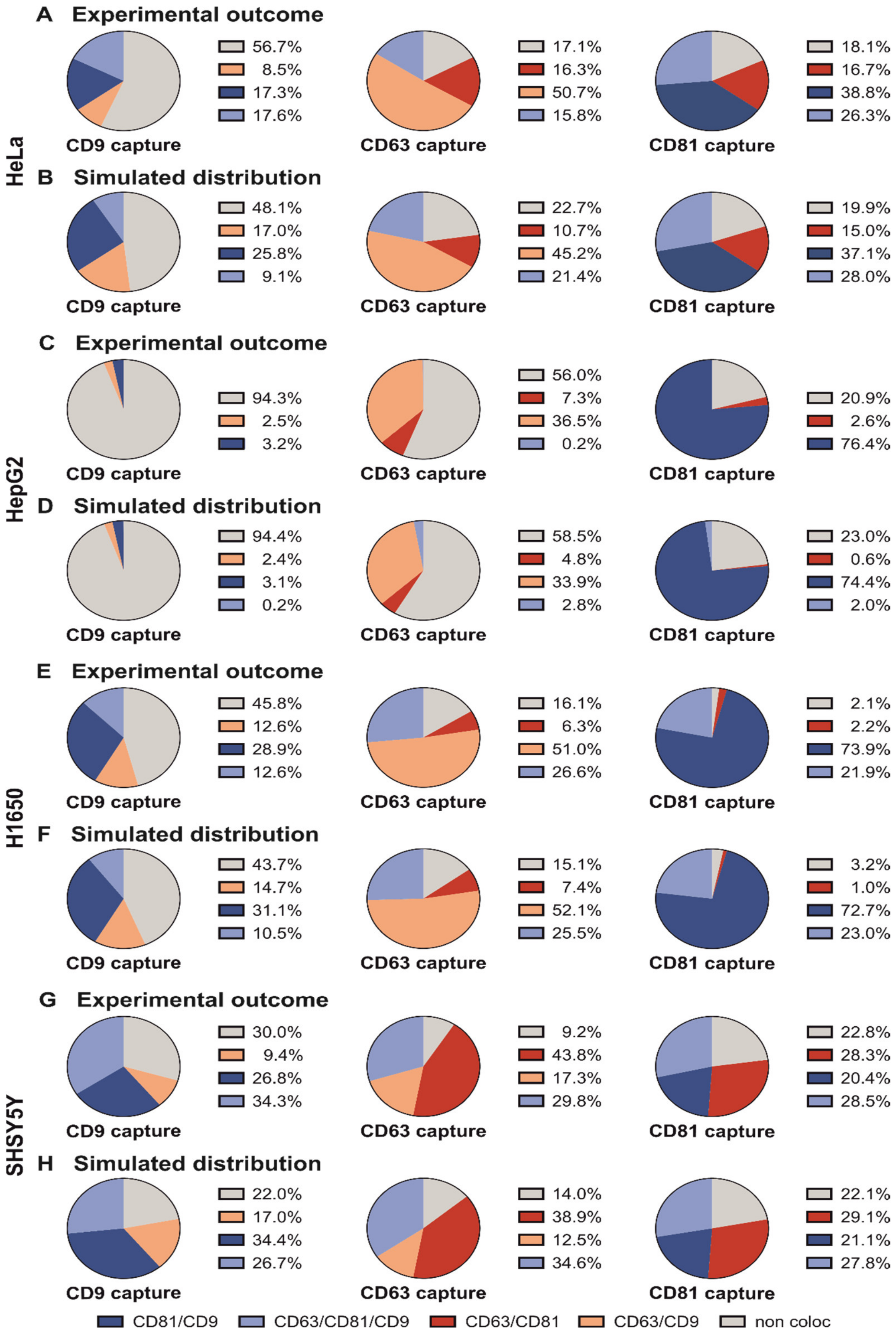

Figure S4. Differences in tetraspanin colocalization between experimental outcome and calculated random distribution of sEV derived from cancer cells. (**A+C+E+G**) Experimentally determined tetraspanin colocalization on the CD9/CD63/CD81 tetraspanin capture spots of HeLa- (**A**), HepG2-

(**C**), H1650- (**E**) and SH-SY5Y-derived (**G**) sEV using the ExoView R100 platform and fluorescent antibodies. (**B+D+F+H**) Simulated random tetraspanin colocalization using decision tree calculation based on experimentally determined data of HeLA- (**B**), HepG2- (**D**), H1650-(**F**) and SH-SY5Y-derived (**H**) sEV. Experimental data are represented as mean of three independent biological experiments with three technical replicates. Predicted random distribution was calculated from the respective experimental data and was generated as indicated.
